# Male Partner Involvement and Development of HIV-exposed Infants in Rural South Africa

**DOI:** 10.1007/s10461-021-03326-5

**Published:** 2021-06-07

**Authors:** Motlagabo Gladys Matseke, Robert A. C. Ruiter, Violeta J. Rodriguez, Karl Peltzer, Deborah L. Jones, Sibusiso Sifunda

**Affiliations:** 1 Department of Work & Social Psychology, Maastricht University, Universiteitssingel 50, 6229 ER Maastricht, Netherlands; 2 HIV/AIDS, STI and TB Research Programme, Human Sciences Research Council, Pretoria, South Africa; 3 Research & Innovation Chief Directorate, The National School of Government, Pretoria, South Africa; 4 Department of Psychology, University of Georgia, Athens, GA USA; 5 Department of Psychiatry & Behavioural Sciences, University of Miami Miller School of Medicine, Miami, USA; 6 Department of Research & Innovation, University of Limpopo, Sovenga, Limpopo, South Africa; 7 ASEAN Institute for Health Development, Mahidol University, Salaya, Thailand

**Keywords:** Male partner involvement, Infant development, HIV-exposed infants, Rural South Africa

## Abstract

Male partner involvement (MPI) during the prenatal and postnatal periods has been proven to have a beneficial effect on infant development. Infants born to HIV seropositive mothers with lacking or no prenatal and postnatal male partner support may be at a higher risk for adverse developmental outcomes. This study examined the effect of MPI on cognitive, communicative, fine, and gross motor development in 160 infants born to HIV seropositive mothers attending Prevention of Mother-to-Child Transmission of HIV (PMTCT) services in rural South Africa. Results of the bivariate logistic regression showed that both prenatal (OR 1.13; 95% CI 1.01, 1.26; p < 0.05) and postnatal MPI (at 12 months) (1.19; 1.07, 1.31; p < 0.005) were associated with risk for delayed gross motor development in HIV exposed infants. Decreased postnatal MPI (0.85; 0.75, 0.98; p < 0.05) was significantly associated with risk for delayed cognitive development. Not living together with a male partner (2.01; 1.06, 3.80; p < 0.05) was significantly associated with risk for delayed cognitive development. In the multivariate logistic regression analysis, decreased postnatal MPI (0.85; 0.75, 0.98; p < 0.05) was significantly associated with risk for delayed cognitive development. On the other hand, postnatal MPI (1.30; 1.12, 1.50; p < 0.005) was associated with risk for delayed gross motor development among HIV exposed infants. Increased MPI can have beneficial effects on infants’ cognitive development. Interventions in PMTCT programs should promote increased prenatal and postnatal MPI to improve cognitive development in HIV exposed infants.

## Introduction

Male partner involvement (MPI) during pregnancy, childbirth and after birth, has been promoted as an effective intervention to improve maternal and infant health outcomes. The beneficial effect of MPI in infant development has been shown in previous studies. In a systematic review of literature, frequent and active paternal engagement predicted a variety of positive infant outcomes including enhanced cognitive development as well as decreased rates of externalizing disorders later in life [1]. A review of infant studies has shown that father-child interactions from as early as 3 months of age may influence children’s cognitive development at 24 months of age [2]. For example, according to this review [2] father-child interactions at 3 months took place at home in a floor-mat setting in which fathers were asked to talk to and play with their infants, for three minutes, as they would normally without the use of toys. At twenty-four months of age, free play and book sessions were applied as father-child interactions. A more positive paternal engagement was associated with a higher Mental Development Index of the Bayley Scales of Infant Development. In comparison to single-parent children, dual-parent children had more mobility, were more active and autonomous, and had more elaborate sociality both in terms of competition and collaboration [3].

Fathers may play a direct role in their children’s development through interaction with them in a variety of ways. They may also play an indirect role in their children’s lives through the emotional and physical support they provide to their children’s mothers [4, 5]. This study focused on the latter type of role, where MPI is reported by women in this study, but no data to show direct infant-father interaction has been collected. A pilot study among HIV infected pregnant women and their infants indicated an association between lack of prenatal MPI and risks for delays in infant cognitive development, gross motor development and receptive communication [6]. There is currently a dearth of research conducted on MPI and infant development in South Africa. This study seeks to explore the relationship between MPI (prenatal and postnatal) and infant development in infants born to HIV infected mothers. Thus, this study aimed to determine whether prenatal and postnatal MPI have an influence on cognitive, communicative, fine and gross motor development in HIV exposed infants in rural South Africa.

## Methods

Prior to the inception of the study, ethical approval was obtained from the University of Miami Miller School of Medicine, the Human Sciences Research Council, and the Department of Health in Mpumalanga Province. Informed consent was obtained from the mothers before enrollment in the original study. In addition, informed consent was also obtained from the same mothers for enrolling their infants for assessment using the Bayley Scales of Infant Development III (BSID-III) screening test.

### Study Design

This study was retrospective and utilized already existing data of 160 HIV positive mothers who participated in a randomized controlled trial in South Africa, aimed at assessing the impact of prenatal and postnatal MPI on adherence to antiretroviral medication and other PMTCT protocols. The trial was conducted in 12 randomly assigned Community Health Centers in Gert Sibande and Nkangala districts in Mpumalanga province, South Africa [7]. As a sub-study in the trial, infant assessments were conducted with 160 infants (of the same mothers) at 12 months of age. Both the datasets from the mothers and infants were merged for the purposes of this study.

### Study Procedures

In the randomized controlled trial, assessments with 160 HIV positive mothers were conducted at five (5) time points, with two assessments occurring during pregnancy (at baseline/8–24 weeks gestation and at 32 weeks gestation) and three assessments after birth (at 3 months, 6 months, and 12 months). For this study, data from assessments conducted at baseline/8–24 weeks (during pregnancy) and 12 months (after birth) were used. The assessments were computerized using the Audio Computer-Assisted Self Interview (ACASI) system to obtain more accurate reporting of sensitive information in comparison with standard interviewing [8]. All materials for maternal assessments were provided in English and local languages (Sesotho, isiZulu) and assessments were approximately 60 min in duration.

Two assessors received a one-week training in the administration of the BSID-III [9] screening test by two licensed clinical psychologists from the University of Miami Miller School of Medicine. Training included role-playing and hands-on practice assessments with infants in the same age range as those in the study. Assessments were administered using the BSID-III screening test to infant participants in the presence and with the consent of their mothers. Assessors were two trained fieldwork personnel and one Bachelor’s degree level research personnel who were fluent in English and the local languages spoken by the mothers (Sesotho and isiZulu). The three assessors had extensive assessment and data collection experience in this setting and had worked with the present sample for 3 years at the time of BSID-III administration. After training, the assessors administered additional practice tests and the two licensed clinical psychologists in the US provided ongoing supervision. Two doctoral students in developmental and clinical psychology reviewed scoring for each of the individual assessments to ensure accuracy.

### Measures

#### Socio-demographics Characteristics

In the first assessment (baseline) during pregnancy, HIV positive women were asked to report on their demographic information such as age, level of education, income, relationship status including living arrangements with male partner, and number of children.

#### Prenatal MPI (at Baseline)

Prenatal MPI was assessed using an adapted version of the Male Involvement Index [7], comprising of 11 items related to participants’ partner involvement in the antenatal period. Questions included “Does your male partner attend antenatal care visits with you?” and “Have you discussed antenatal HIV prevention for your baby with your male partner?” Participants responded to each item with either a Yes (1) or No (0). Total possible scores ranged from 0 to 11, with higher scores representing more MPI. Cronbach’s alpha was adequate at 0.83, at baseline.

*Postnatal MPI (at 12 months)* comprised of similar items related to participants’ partner involvement in the postnatal period at 12 months of infant age. Questions included “Does your male partner attend infant care visits with you?” and “Does your male partner know your infant care appointment time?”. Participants responded to each item by either a Yes (1) or No (0), and scores ranged from 0 to 11. Cronbach’s alpha was adequate at 0.82.

*Disclosure of HIV serostatus to partner* was assessed by asking the question “Have you disclosed your HIV status to your spouse/partner?” Response options were either a “Yes” or a “No”. This question is one of the items of the adapted version of the Disclosure Scale [10]. The baseline (prenatal) and 12 months postnatal measures were used in this study. However, only the postnatal measure was used as it yielded a stronger model in the multiple regression analysis.

#### Infant Development

The BSID-III [9] screening test was used to evaluate five domains of infant development at 12 months of age, that is, cognitive, receptive communication, expressive communication, fine motor, and gross motor skills. The BSID-III has been previously used in South Africa to assess infants at 3, 6, 9, and 12 months of age [11]. An earlier version (second edition) of the BSID has also been used in children of a mean age of 15.8 months in South Africa [12], which was administered by a trained researcher fluent in English and a local language, as in this study. Raw scores were dichotomized into ‘competent’ and ‘at emerging risk or at risk’, using standard validated cut-offs [9] to (a) maximize predictive power given the small sample size, (b) use a standard metric across infants, and (c) identify infants who normally would be classified to be at risk in clinical settings and in need of an intervention. Neurodevelopmental testing guidelines recommend dichotomization and advice against the use of 1-SD cut-offs [13]. This assessment has previously been used in South Africa to assess infants at 3, 6, 9, 12, and 16 months of age without adaptation [12]. As in previous studies, adaptation or translation of the assessment was not deemed necessary due to the age of the infants and to maximize the generalizability of the findings [14–16]. An assessor was available to explain the procedures and translate instructions for the mothers when necessary.

### Data Analysis

Statistical Package for Social Sciences (SPSS version 24.0, IBM, Armonk, NY, USA) was used to analyze the data. Univariate analyses were used to describe the sample, and 95% confidence intervals (CI) were calculated using 1000 bootstrap samples. Bivariate logistic regression analyses were used to assess bivariate associations between domains of infant development (competent or at emerging risk/at risk) and maternal demographic and psychosocial characteristics, as well as MPI.

A series of multivariate logistic regression models were then built to examine cognitive,receptive and expressive communication, and fine and gross motor development as outcomes,and pre- and postnatal MPI as independent variables. All models accounted for the effect of using two trained assessors to administer the BSID-III assessments and infant HIV serostatus. Because infant age and development are closely related as part of natural developmental progression, the inclusion of age as a statistical control was redundant. As such, given the total possible scores for each of the subscales depended on infant age, in months and days, and mean infant age was 13.94 months for this study. Raw scores were dichotomized into “competent” versus “at emerging risk”/ “at risk for developmental delay”, which allowed all infants to be on the same scale regardless of age [9]. Demographic and psychosocial variables associated with cognitive, receptive and expressive communication, and fine and gross motor development at p < 0.10 in bivariate analyses were also included as independent variables in all models.

## Results

### Demographic Characteristics

Demographic characteristics of the sample are shown in Table 1. The mean age of women in this study was 28.96 years (SD = 5.58). Most (52.3%) of the women were not married and lived separately from the partners or fathers of their children, 68.4% had less than grade 12, and 68.4% had a monthly income of less than R1000. Half (50%) of the women had disclosed their HIV status to their male partner at 12 months postnatal. The mean age of infants in this study was 13.94 months (SD = 3.01).

**Table 1:** Demographic and psychosocial characteristics of infant-mother dyad participants (N = 160)

Characteristic	Mean (SD) N (%)
Mother	
Age	28.96 (5.58)
Educational attainment	
Grade 0–11	47 (68.4%)
Grade 12 or more	22 (31.6%)
Monthly household income (rand)	
< 999 (~ $76)	47 (68.4%)
1000 or more	22 (31.6%)
Relationship Status	
Unmarried, living separately	81 (52.3%)
Unmarried, living together	45 (29.0%)
Married	29 (18.7%)
Months since HIV diagnosis	26.2 (38.12)
Disclosure of serostatus to male partner (at 12 months postnatal)	
No	60 (50.0%)
Yes	60 (50.0%)
Male involvement, baseline (index)	7.08 (3.02)
Male involvement, at 12 months (index)	6.38 (3.46)
Infants	
Age	13.94 (3.01)
Infant development (Bayley’s scale) scores	
Cognitive	2.45 (0.69)
Receptive communication	2.46 (0.76)
Expressive communication	2.28 (0.76)
Fine motor	2.38 (0.70)
Gross motor	2.23 (0.77)

### Prevalence of MPI and Infant Developmental Delays

Table 2 shows the prevalence of prenatal and postnatal MPI in the sample of mothers, and the prevalence of developmental delays in the sample of infants. The mean MPI score was 7.08 (SD = 3.02) prenatally and 6.38 (SD = 3.46) postnatally as shown in Table 1. Table 2 indicates that ten (10) out of eleven (11) prenatal and postnatal MPI activities were reported by over half (50%) of the women in this study.

**Table 2:** Male partner involvement and infant development at 12 months (N = 160)

Items	N (%)
Cognitive	
At risk	18 (11.3)
Emerging risk	52 (32.5)
Competent	90 (56.3)
Receptive communication	
At risk	29 (18.2)
Emerging risk	56 (35.2)
Competent	74 (46.5)
Expressive communication	
At risk	20 (12.6)
Emerging risk	59 (37.1)
Competent	80 (50.3)
Fine motor development	
At risk	20 (12.6)
Emerging risk	59 (37.1)
Competent	80 (50.3)
Male partner involvement (prenatal**)**	
Male partner attends antenatal care visits with you	42 (27.1)
Male partner knows your antenatal appointment time	115 (74.2)
Discussed antenatal HIV prevention for your baby with your male partner	103 (66.5)
Male partner supports your antenatal visits financially	131 (84.5)
Male partner knows what happens in the antenatal clinic	92 (59.4)
After testing for HIV, partner asked to take an HIV test	108 (69.7)
Told partner that you were told to take ARV drugs (HIV medication)	104 (67.1)
Discussed feeding options for your baby with your male partner	107 (70.3)
Discussed the place of delivery for the baby with your male partner	96 (61.9)
Discussed testing your baby for HIV with your male partner	82 (52.9)
Discussed condom use with your male partner	116 (74.8)
Male partner involvement (postnatal**)**	
Male partner attends infant care visits with you	70 (44.3)
Male partner knows your infant care appointment time	113 (71.5)
Discussed HIV prevention for your baby with your male partner	99 (62.7)
Male partner supports your infant care visits financially	125 (79.1)
Male partner knows what happens in the infant care clinic	84 (53.2)
After tested for HIV, male partner was asked to take an HIV test	96 (60.8)
Told partner that you were told to take ARV drugs (HIV medication)	110 (69.6)
Discuss feeding options for your baby with your male partner	100 (63.3)
Discussed testing your baby for HIV with your male partner	100 (63.3)
Discussed condom use with your male partner	111 (70.3)

Table 2 also shows that about 11.3% of the infants were at risk and 32.5% at emerging risk for delays in cognitive development. With regards to receptive communication, 18.2% and 35.2% of the infants were at risk and at emerging risk for developmental delays, respectively. Twelve-point six percent (12.6%) were at risk and 37.1% at emerging risk for developmental delays with regards to expressive communication. About 12.6% of the infants were at risk for delays in fine motor development, while 37.1% were at emerging risk. Twenty-point-nine percent (20.9%) were at risk and 35.4% at emerging risk for delays in gross motor development.

### Bivariate Associations with Risk for Delays in Cognitive, Expressive Communication, Receptive Communication, Fine and Gross Motor Development Among Infants

Table 3 shows the results of the unadjusted logistic regression analysis between study variables. Not living together with male partner (OR 2.01; 95% CI 1.06, 3.80; p < 0.05) and decreased/lack of postnatal MPI (0.88; 0.80, 0.97; p < 0.05) were significantly associated with risk for delayed cognitive development. Both prenatal MPI (1.13; 1.01, 1.26; p < 0.05) and postnatal MPI (1.19; 1.07, 1.31; p < 0.005) were significantly associated with delayed gross motor development among the infants. Receptive communication, expressive communication, and fine motor development were not significantly associated with any of the independent variables.

**Table 3:** Unadjusted logistic regression models predicting risk for delayed cognitive, expressive communication, receptive communication, and fine and gross motor development (N = 160)

Predictor	Cognitive OR (95% CI)	Receptive communication OR (95% CI)	Expressive communication OR (95% CI)	Fine motor OR (95% CI)	Gross motor OR (95% CI)
Unmarried, not living together (ref = married or unmarried, living together)	2.01 (1.06, 3.80)*	1.11 (0.58, 2.10)	1.27 (0.68, 2.39)	1.19 (0.64, 2.23)	1.21 (0.64, 2.39)
Household income (ref = below the poverty level, < 600)	0.93 (0.49, 1.74)	1.24 (0.65, 2.36)	1.70 (0.90, 3.20)	0.80 (0.43, 1.50)	1.07 (0.57, 2.01)
Number of children (ref = no children)	0.70 (0.31, 1.58)	1.83 (0.82, 4.07)	1.65 (0.73, 3.74)	1.67 (0.75, 3.75)	1.60 (0.69, 3.71)
Disclosure to partner at baseline (ref = not disclosed)	1.44 (0.75, 2.75)	0.97 (0.50, 1.89)	1.11 (0.58, 2.13)	1.37 (0.72, 2.64)	1.86 (0.95, 3.65)
Disclosure to partner at 12-months (ref = not disclosed)	0.58 (0.28, 1.20)	0.93 (0.45, 1.93)	0.90 (0.43, 1.86)	0.84 (0.41, 1.73)	0.70 (0.34, 1.47)
Baseline MPI	0.96 (0.87, 1.07)	0.97 (0.87, 1.08)	0.97 (0.88, 1.08)	1.08 (0.97, 1.20)	1.13 (1.01, 1.26)*
12-month MPI	0.88 (0.80, 0.97)*	0.94 (0.85, 1.03)	1.07 (0.98, 1.17)	1.05 (0.96, 1.15)	1.19 (1.07, 1.31)**

*p < 0.05, **p < 0.01, ***p < 0.001

### Multivariate Associations with Risks for Delays in Cognitive, Expressive Communication, Receptive Communication, Fine and Gross Motor Development Among Infants

As indicated in Table 4, decreased MPI during pregnancy (AOR 0.95; 95% CI 0.81, 1.11; p > 0.05) and at 12 months postnatal (0.85; 0.75, 0.98; p < 0.05) were associated with delayed cognitive development among HIV exposed infants. However, this association was significant only for decreased MPI at 12 months postnatal. On the other hand, MPI during pregnancy (1.08; 0.91, 1.29; p > 0.05) and at 12 months postnatal (1.30; 1.12, 1.50; p < 0.005) was associated with delayed gross motor functioning in HIV exposed infants. This association was significant only for MPI at 12 months postnatal. Prenatal MPI [(0.97; 0.87, 1.08; p > 0.05)] [(0.97; 0.88, 1.08; p > 0.05)], [(1.08; 0.97, 1.20; p > 0.05)] was not significantly associated with delays in communicative and fine motor development (respectively) in HIV exposed infants. Similarly, postnatal MPI [(0.94; 0.85, 1.03; p > 0.05)], [(1.07; 0.98, 1.17; p > 0.05)], [(1.05; 0.96, 1.15, p > 0.05)] was not significantly associated with delays in communicative and fine motor development (respectively) in HIV exposed infants. Non-disclosure of HIV status at 12 months postnatal (0.21; 0.07, 0.61; p < 0.01) was significantly associated with delayed gross motor development in HIV exposed infants.

**Table 4:** Adjusted logistic regression models predicting risk for delayed cognitive, expressive communication, receptive communication, and fine and gross motor development (N = 160)

Predictor	Cognitive^a^ OR (95% CI)	Receptive communication^b^ OR (95% CI)	Expressive communication^c^ OR (95% CI)	Fine motor^d^ OR (95% CI)	Gross motor^e^ OR (95% CI)
Unmarried, not living together (ref = married or unmarried, living together)	0.61 (0.26, 1.45)	0.91 (0.39, 2.11)	0.68 (0.29, 1.63)	0.58 (0.25, 1.37)	0.58 (0.23, 1.50)
Household income (ref = below the poverty level, < 600)	0.89 (0.39, 2.00)	0.92 (0.42, 2.01)	1.92 (0.86, 4.30)	0.79 (0.36, 1.74)	0.87 (0.36, 2.10)
Number of children (ref = no children)	0.68 (0.24, 1.92)	1.23 (0.47, 3.20)	1.31 (0.50, 3.49)	1.68 (0.62, 4.52)	1.58 (0.52, 4.80)
Disclosure to partner at baseline (ref = not disclosed)	2.53 (0.95, 6.75)	1.03 (0.41, 2.58)	1.35 (0.53, 3.47)	0.99 (0.40, 2.49)	1.24 (0.45, 3.34)
Disclosure to partner at 12-months (ref = not disclosed)	1.04 (0.41, 2.61)	1.39 (0.57, 3.39)	0.63 (0.25, 1.56)	0.64 (0.26, 1.60)	0.21 (0.07, 0.61)**
Baseline MPI	0.95 (0.81, 1.11)	0.96 (0.82, 1.12)	0.96 (0.82, 1.12)	1.04 (0.89, 1.21)	1.08 (0.91, 1.29)
12-month MPI	0.85 (0.75, 0.98)*	0.93 (0.82, 1.05)	1.12 (0.98, 1.27)	1.10 (0.97, 1.24)	1.30 (1.12, 1.50)**

*p < 0.05, **p < 0.01, ***p < 0.001

^a^Hosmer and Lemeshow χ^2^ = 6.48, p = 0.594; Nagelkerke *R*^2^ = 0.162

^b^Hosmer and Lemeshow χ^2^ = 14.50, p = 0.070; Nagelkerke *R*^2^ = 0.032

^c^Hosmer and Lemeshow χ^2^ = 13.25, p = 0.104; Nagelkerke *R*^2^ = 0.071

^d^Hosmer and Lemeshow χ^2^ = 5.93, p = 0.655; Nagelkerke *R*^2^ = 0.053

^e^Hosmer and Lemeshow χ^2^ = 6.00, p = 0.647; Nagelkerke *R*^2^ = 0.214

## Discussion

This study examined the influence of MPI during and after pregnancy on cognitive, communicative, fine and gross motor development in infants born to HIV seropositive mothers attending PMTCT services in rural South Africa. While the prevalence of MPI (during and after pregnancy) reported by women in this study was high, the prevalence of risk/emerging risk for delays in cognitive, communicative, and motor development among infants was also high.

All infants in this study were exposed to HIV which partly accounts for the high prevalence of developmental delays [17]. Reasons for developmental delays among HIV exposed infants are multifactorial, but the result of this study provides evidence that infants whose mothers received less or no MPI during pregnancy or during the postnatal period were more likely to be at risk for delays in cognitive and gross motor development. These reasons, of risks for developmental delays among HIV exposed infants, include increased risk of HIV infection to the infant where mothers did not disclose their positive HIV status to their male partners.

In the final analysis, decreased/lack of postnatal MPI and not living together with male partners were associated with risk for delayed cognitive development. This suggests that infants whose mothers did not share a home with male partners were more likely to have delayed cognitive development. This suggestion makes sense since sharing a home with a male partner is likely to provide an opportunity for frequent paternal engagement with infants which in turn enhances their cognitive and motor development. Previous literature has shown that frequent, active and regular paternal engagement with the child from infancy has been associated with enhanced infant cognitive development among other positive outcomes [1, 2]. Rodriguez et al. [6] have shown an association between not living together with male partners during pregnancy and risk for delays in infant cognitive development, gross motor development and development in receptive communication in infants born from HIV infected women.

Contrarily, postnatal MPI was associated with risk for delayed gross motor development among the HIV exposed infants. This finding was unexpected due to the already stated view that provided justification for the association between MPI and cognitive development in infants. This issue warrants further investigation.

Furthermore, non-disclosure of HIV status at 12 months postnatal was significantly associated with risk for delayed gross motor development in HIV exposed infants. This finding supports notions that non-disclosure of HIV status by pregnant women to male partners will less likely lead to the adoption of safer sexual behaviors and risk of re-infection if both partners are infected [18]. As such, adopting safer sexual behaviors will minimise the risk of HIV transmission to unborn babies during pregnancy and to infants after birth. Findings and deliberations by previous studies have stated the importance of HIV status disclosure by pregnant women to their male partners [6, 19, 20]. It is crucial for pregnant women to disclose their HIV status to sexual partners to enable the adoption of safer sexual behaviours and to minimise the risk of HIV transmission to the unborn babies and after birth [18].

This paper provides evidence that male partners (fathers) can indirectly influence infants’ development through positive involvement in the life of the mother during and after pregnancy. According to Rosenberg and Wilcox [5] fathers can have an indirect influence on their children’s development through the quality of their relationship with the mother of their children. Fathers who have good relationships with the mother of their children are more likely to be involved and to spend time with their children and as a result have children who are psychologically and emotionally healthier [5]. Similarly, mothers who feel affirmed by the fathers of their children and enjoy the benefits of happy relationships are more likely to be better mothers, leading to positive infant development outcomes.

For pregnant mothers, living together with male partners will more likely create an enabling environment for positive interaction that is likely to be conducive in-utero, ultimately enhancing positive infant development. However, pregnant mothers living separately from their male partners may also receive different types of available support such as financial and emotional support [20] that may enhance positive infant development outcomes after birth.

The influence of MPI on receptive communication, expressive communication, fine and gross motor development in infants should be further explored in future studies.

## Limitations

Some limitations were noted in this study. This study relied on self-reported MPI by mothers rather than self-reports from fathers. As such, there was no evidence of direct father-child interaction or involvement in this study. Although it may be challenging in the real world, future studies could therefore explore the effect of direct father-child interaction or involvement on the different domains of infant development. Also, since this was a non-experimental study future studies could experimentally explore the effect of MPI on HIV exposed infants’ cognitive, expressive communication, receptive communication, fine and gross motor development.

There may be other important factors, moderators, and mediators influencing infant development that are not assessed and that could have uncovered more complex relationships and pathways between MPI and infant development.

## Conclusion

The results of this study provide important information for consideration by policymakers in the maternal and child health programs. Infants who participated in this study were all exposed to HIV (i.e. born from HIV infected mothers) and as such, were all at risk for developmental delays, which may account for the high prevalence of delayed development in this sample. The high levels of delayed cognitive, communicative, fine and gross motor development among infants in this study are of great concern and thus warrants appropriate interventions. The low levels of MPI reported by women may need to be improved. Increased MPI can have beneficial effects on infant cognitive development as suggested by the results of this study. Interventions in PMTCT should therefore promote increased prenatal and postnatal MPI to improve cognitive development in HIV exposed infants.
